# Operative Techniques for Cervical Radiculopathy and Myelopathy

**DOI:** 10.1155/2012/916149

**Published:** 2011-12-13

**Authors:** C. Moran, C. Bolger

**Affiliations:** National Centre for Neurosurgery, Beaumont Hospital, Dublin, Ireland

## Abstract

The surgical treatment of cervical spondylosis and resulting cervical radiculopathy or myelopathy has evolved over the past century. Surgical options for dorsal decompression of the cervical spine includes the traditional laminectomy and laminoplasty, first described in Asia in the 1970's. More recently the dorsal approch has been explored in terms of minimally invasive options including foraminotomies for nerve root descompression. Ventral decompression and fusion techniques are also described in the article, including traditional anterior cervical discectomy and fusion, strut grafting and cervical disc arthroplasty. Overall, the outcome from surgery is determined by choosing the correct surgery for the correct patient and pathology and this is what we hope to explain in this brief review.

## 1. Introduction

Cervical spondylosis is a common pathology, and the surgical treatment of the resulting radiculopathy, myelopathy, or myeloradiculopathy has evolved over the past century. The basic aim of all techniques is to decompress the affected neural structure. Advances in fixation techniques [1–3] and motion-preserving options [4–7] are more recent elements of this evolution. Once the decision is made to manage the patient operatively the principal decision is whether to choose the ventral or the dorsal approach. In cervical spondylosis several variables including the location of pathology (ventral, dorsal, circumferential); extent of pathology (limited to interspace, extensive behind vertebral body); the number of levels affected; the presence of instability or the presence of kyphotic deformity require consideration.

In general, any procedure chosen should decompress the affected spinal cord or nerve roots, maintain or restore stability, and correct or prevent kyphotic deformity.

## 2. Dorsal Decompression 

A range of posterior surgical procedures exist, including laminectomy, laminoplasty, and laminectomy with posterior fusion. Until the 1960's the traditional way to decompress the cervical spine in spondylotic patients was via a dorsal approach and a decompressive laminectomy. This surgery effectively enlarges the spinal canal area, allowing the spinal canal to drift away from ventral compression, however, while doing this it also destabilizes the dorsal structures and can lead to progressive kyphotic deformity.

## 3. Laminectomy

A high speed drill is used to create a gutter, through the outer cortical bone and cancellous bone to the thin inner cortical bone at the junction of the lamina and the medial aspect of the lateral mass. Using a 1 mm Kerrison rongeur the lamina and ligamentum flavum is then transacted laterally. Two Kocher clamps are then used to remove the dorsal elements en bloc. In patients with loss of lordosis or abnormal segment motion, at this stage once decompression is completed, then instrumentation can be performed. Many wiring techniques are not suitable as the posterior elements have been removed. Options for fixation include interfacet wiring, which is unpopular due to postoperative pain caused by violating an intact facet joint. Lateral mass fixation techniques are the most popular and include fixation of a plate or rod to the lateral masses using screws [8–11].

## 4. Laminoplasty

Cervical laminoplasty, posterior decompression of the cord with reconstruction of the laminae, was a technique developed in Asia from the 1970's onwards, Hattori first described the Z-shaped laminoplasty [12–14]. The rationale was to leave the dorsal stabilizing structures in situ, allowing for fusion after decompression and therefore prevent the subsequent development of kyphotic deformity [15–18]. It is today used for multiple level spondylosis without kyphosis. In the Hirabayashi method osteotomies of cervical lamina to be included are performed on one side to create an “open” and a “hinged” side. The open side is chosen according to the most symptomatic side and whether or not foraminotomies will also be performed. High speed drill creates troughs at the level of the lamina-facet junction. The procedure ranges from the laminar level one above to the level below the stenotic site. Both the outer and inner cortical margins are drilled through on the “open” side, while the inner cortical margin is kept intact on the “hinge” side. After the excision on the open side is completed the spinous processes and laminae are pushed laterally as if opening a door, any adhesions to the dura are divided. The ligamentum flavum and deep muscles around the facets of the hinged side are then supported by sutures to prevent closing of the laminae. Alternatively allograft tricortical iliac crest or manufactured bone spacers can be placed in the open-door portion of the laminoplasty and stabilized using 2.0 mm titanium miniplates. Nakano et al. [18] reviewed patients who had undergone laminoplasty for OPLL with more than 10 years of followup. Long-term outcomes were good, with 64% mean neurological recovery in the first 10 years and 60% at the final followup. 

## 5. Ventral versus Dorsal Decompression

Ventral decompression is most appropriate in patients who have ventral compression limited to one or two vertebral body levels, but in the setting of multilevel disease decompression from the front is complicated by lower fusion rate, adjacent level disease, and hardware failure [19]. The degenerative process includes hypertrophic changes circumferentially, and enlarged facet joints and thickened ligaments can contribute significantly to the narrowing of the spinal canal diameter throughout the entire cervical spine. Ventral approaches are often inadequate, especially when long-segment decompression is required. Dorsal approaches, including laminectomy with or without fusion and laminoplasty, provide surgical expansion to the spinal canal. 

Sagittal balance is an important factor in determining whether a dorsal approach is suitable treatment; if the c-spine is kyphotic the cord will remain draped over the ventral compressive elements after dorsal decompression and the surgery will be unsuccessful.

Advantages of dorsal surgery include less surgical effort and time to decompress multiple levels and less frequent need for instrumentation and fusion, thereby helping decrease the chance of adjacent level disease, and it also affords direct visualisation of nerve roots when decompressing, and there is little risk to anterior neck structures such as the recurrent laryngeal nerve and the oesophagus.

## 6. Anterior Cervical Discectomy and Fusion

The ventrolateral approach for decompression of the cervical spine and nerve roots has become a well-practised technique among spinal surgeons. It was first described by Cloward [20] and Smith and Robinson [21] over forty years ago and has evolved to one of the most popular spinal surgery operations. The approach allows for safe and direct decompression of the spinal cord at the site of compression. The technique is used to treat radiculopathy due to disc herniation and osteophyte formation as well as cervical myelopathy or myeloradiculopathy. The most common levels affected are C5 to C6, then C6 to C7, then C4 to C5 in order of frequency.

The operation is performed in a supine position, with the neck in a moderately hyperextended position. The approach is routinely from the right; the incision can be centered on anatomical landmarks, including the hyoid at C3, the thyroid cartilage at C4, and the cricoid at C6, alternatively fluoroscopy can be obtained using a metal object taped to skin. A transverse incision is preferred, in a skin crease for good cosmesis, the incision is 5-6 cm extending to the medial border of the sternocleidomastoid. Dissection is performed down to platysma, this is then sharply incised transversely across the length of the skin incision and elevated. The cervical fascia is then opened vertically just anterior to the sternocleidomastoid, blunt dissection is then used to separate the soft-tissue plane between the lateral aspect of the laryngeal strap muscles and the medial aspect of the SCM. An avascular plane is developed down to the vertebral bodies by retracting the trachea and oesophagus medially and the carotid sheath laterally, 7the carotid artery is palpated for behind the sternocleidomastoid. Close attention should be paid to avoid dividing any structure crossing from the carotid sheath medially. The prevertebral fascia is then opened in the midline. A spinal needle is placed in the disc space and the level is checked with a lateral radiograph. Once the correct level is confirmed the longus colli muscles are cauterised, using bi-polar diathermy and reflected laterally; dissection continues out to the uncinate processes and self-retaining retractors are then placed beneath the medial edges of the reflected longus colli muscles. These retractors should not be displaced for the remainder of the surgery. Vertebral body posts are then placed in the vertebral bodies above and below the disc to be removed and the bodies are distracted gently. The anterior longitudinal ligament is removed. The annulus fibrosus is incised and superficial disc and cartilaginous end plates can then be removed, using rongeurs, drill, and curettes. Posts inserted into the vertebral body at this stage can be spread again, thus increasing the disc height. The remainder of the disc is then removed, as well as the posterior annulus and osteophytic ridges. The removal of the posterior longitudinal ligament is also routinely performed. Each neural foramen is cleared and palpated afterwards to ensure adequate nerve root decompression. The interbody graft is then placed after the adjacent end plates have been drilled to promote fusion. The graft is then tapped into position and the distraction is released.

## 7. Fusion Techniques

There are three different types of anterior cervical fusions, described by Cloward [20] Smith and Robinson [21], and Simmons et al. [22] which employ bone-grafting techniques. Iliac corticocancellous graft is used in the Cloward technique; these grafts are typically 12–16 mm in diameter and 10 to 14 mm in height, and the bone is seated into the softer cancellous portion of the midvertebral body. A horseshoe-shaped graft is used in the Smith Robinson technique, and less bony resection is performed in this operation, as opposed to Cloward, where the lateral exposure is greater, the graft is seated on the stronger subchondral bone end plate.

## 8. Artificial Cervical Disc Arthroplasty

Alternatives to anterior cervical discectomy and fusion have been developed that attempt to address some of the kinematic and biomechanical issues associated with fusing two spinal levels. It has been shown that twenty-five percent of patients undergoing cervical fusion will have new onset symptoms within ten years of that fusion due to degenerative changes at adjacent levels [23]. Total intervertebral disc replacement was introduced to preserve motion and restore disc height after removing the pathology. The Bryan cervical disc prosthesis was first introduced in the USA in 2002. It was used for the treatment of both radiculopathy and myelopathy, and in recent studies comparing the two for single-level disease and comparing artificial disc versus fusion have shown both groups to have comparable improvement in all outcome measures [24].

## 9. Cervical Corpectomy and Strut Grafting

Cervical corpectomy can be used for a variety of spinal disorders, including infection, neoplastic disease, and trauma, but it is most commonly used for multilevel cervical spondylosis. Single-level vertebrectomy can be carried out on patients with signs and symptoms of myelopathy, who are found on imaging to have spinal cord compression by osteophyte formation and soft-disc herniation at two adjacent levels. Three-level disease and compression can be treated by a two-level vertebrectomy or a multilevel laminectomy. A vertebrectomy should be performed if there is straightening of the spine, as progressive kyphosis is likely after laminectomy or kyphotic deformity as the cord is unlikely to move posteriorly away from the compression in the presence of kyphosis. When compression stretches four motion segments a posterior decompression is preferred, but again, in situations of kyphotic deformity a three-level vertebrectomy is suitable, and then supplemented with a posterior instrumentation to decrease the risk of graft and plate dislodgement. OPLL also usually requires at least a one-level vertebrectomy.

Regarding the operative procedure, the same approach as the ACDF described above is used. Once the correct level is confirmed the anterior longitudinal ligament over the disc spaces above and below the level to be resected is incised; the most anterior portions of the underlying discs are then removed, as is the anterior longitudinal ligament covering the front of the vertebrae. The extent of bone to be removed is then marked, the width of bony resection is usually 18 mm, but can be decreased to 15 mm at C4 or C5 level surgery to decrease the incidence of C5 nerve root dysfunction [24]. An operating microscope is used as bony resection proceeds using a diamond burr; care is taken once the superficial portion of the body is removed as the vertebral artery lies in the middle third of the AP diameter of the vertebral body. As bony resection proceeds, the end plates of the adjacent vertebrae are also resected. The posterior longitudinal ligament is then opened taking care to lift it away from the dura, and Kerrison rongeurs are then used to resect it as far as the bony exposure. Bony reconstruction can be accomplished using an allograft, autograft, or cage system. The graft should be 2 mm longer than the length of the vertebrectomy, the AP depth is 13 mm. Distraction pins are used above and below in order to insert the construct; using a graft holder it is then hammered into place until it is flush with the anterior aspect of the adjacent vertebral bodies.

Anterior plating is then performed, and the screws are placed under fluoroscopic guidance, as this ensures accurate screw placement avoiding both the graft and the adjacent disc spaces; engagement of the posterior cortex has been shown unnecessary [25, 26].

## 10. Multiple Level Discectomy and Fusion versus Corpectomy 

Cervical corpectomy is an alternative technique for the removal of ventral compressive pathology and stabilization of the cervical spine, and it allows for decompression behind the midportion of the vertebral body. It has been shown that by using ventral instrumentation in two-level discectomy, the fusion rate is comparable with single-level corpectomy [27, 28]. Comparison between three-level discectomy with two-level corpectomy also showed similar rates of fusion. Graft displacement after corpectomy is proportionate to graft length and is increased with fusion ending at the C7 vertebral body. After placing a long corpectomy graft under distraction there tends to be a general straightening of the spine, however, multiple level discectomy and fusion allows for increasing the ventral column height and restoration of lordosis by pulling the vertebral body segments toward the lordotic ventral instrumentation. Also multilevel discectomy and fusion provides more fixation points to hold the construct rigidly, compared with corpectomy and strut grafting which has only two points of fixation and allows for more translational movement [29].

## 11. Multiple Level Discectomy versus Dorsal Procedures

Cervical radiculopathy is caused by compression at multiple levels; if lordosis is maintained and the compressive pathology is primarily foraminal then dorsal foraminotomies are a reasonable alternative. However dorsal foraminotomy cannot correct kyphosis and may predispose to it.

Cervical Myelopathy is best treated using a dorsal approach when the compression is primarily dorsal, that is, congenital stenosis; dorsal fusion should be considered if there is a straightening of the spine, kyphosis, or instability.

## 12. Minimally Invasive Surgery and Laminoforaminotomy

Dorsal surgical approaches for the management of cervical spondylosis are well established and in recent years have undergone a host of modifications in order to make the surgeries minimally invasive. The rationale behind these advances is to cause less tissue injury on exposure, thereby decreasing postoperative pain, but most importantly the aim is to decompress the neural structures with minimal disruption of dorsal structures, thereby preserving motion and decreasing adjacent level disease and the incidence of postdecompression kyphosis.

Laminoforaminotomy can be used for nerve root and central canal decompression [30]. Patients with a unilateral monoradiculopathy are best candidates. This is performed using a tubular retractor system, (Met Rx system), a microendoscope, with the patient in a sitting position. Fluoroscopy is used to mark a point 1.5 cm off the midline directly over the desired disc space. A stab incision is made in the skin and a guide pin is docked onto the superior facet at the desired level using fluoroscopic guidance. The stab incision is then widened to 2 cm and the underlying fascia is divided sharply with scissors. The first tissue dilator is then passed over the guide pin and it is removed, further dilators are passed in sequence, the final port is the working channel to which the endoscope is attached. A high speed drill can be used to thin out the medial aspect of the superior facet and a Kerrison rongeur used to perform a foraminotomy. In disc herniation, the nerve root can be gently retracted using a suction tip, allowing space to explore the ventral epidural space. Any epidural plexus bleeding is controlled with electrocautery and hemostatic agents. Two adjacent levels can be decompressed through a single incision by centering the initial incision halfway between the neural foramina.

## 13. Facet Distraction

Recently the technique of facet distraction was published as a treatment option for single or multilevel cervical spondylotic radiculopathy and myelopathy [31]. In this study the authors described facet distraction by manual implantation of metal spacers within the articular cavity after wide removal of the articular cartilage; structural changes resulting from these spacers included an increase in interlaminar and interspinous process distance and restoration of buckled ligaments of the region as well as an increase in spinal canal diameter.

## 14. Summary

Both cervical spondylosis and its most common clinical manifestations, radiculopathy and myelopathy, are common clinical conditions. It occurs in the aging population as a result of disc degeneration, consequent degenerative changes of the uncovertebral joints, ligamentum flavum, and facet complex. Surgical outcome is dependent on selecting the appropriate treatment for the appropriate patient and pathology. In recent years much effort has been focused on modifying the dorsal approaches in an effort to achieve similar results with less tissue injury and less postoperative pain. However, despite the numerous surgical options available, the optimal procedure can still be difficult to choose. 
